# The protective role of *Lactobacillus reuteri* in a DSS-induced ulcerative colitis mouse model

**DOI:** 10.1371/journal.pone.0335942

**Published:** 2025-11-06

**Authors:** Mohsen Nazari, Rezvan Goodarzi, Atefeh Yazdan Panah, Iraj Amiri, Yasser Bagheri, Babak Asghari

**Affiliations:** 1 Department of Microbiology, School of Medicine, Hamadan University of Medical Sciences, Hamadan, Iran; 2 Research Center for Molecular Medicine, Institute of Cancer, Avicenna Health Research Institute, Hamadan University of Medical Sciences, Hamadan, Iran; 3 Anatomy Department, School of Medicine, Hamadan University of Medical Sciences, Hamadan, Iran; 4 Immunology Department, Faculty of Medicine, Golestan University of Medical Sciences, Gorgan, Iran; Northwest Institute of Plateau Biology Chinese Academy of Sciences, CHINA

## Abstract

Ulcerative colitis (UC) is a chronic inflammatory disease of the colon, marked by symptoms such as mucosal ulceration, diarrhea, and abdominal pain. The dextran sodium sulfate (DSS)-induced murine model replicates clinical and histological features of UC and is widely used to explore potential treatments. *Lactobacillus reuteri* has emerged as a promising probiotic due to its immunomodulatory and anti-inflammatory properties. This study investigates the effects of *L. reuteri* supplementation on histopathology, inflammatory cytokines, intestinal barrier function, and short-chain fatty acid (SCFA) production in DSS-induced colitis. Male Balb/C mice were divided into control, DSS-induced colitis, and DSS + *L. reuteri* treatment groups. The colitis model was established with 3% DSS in drinking water for seven days, and mice in the treatment group received 10^10^ CFU of *L. reuteri* daily. Disease activity index (DAI), colon length, myeloperoxidase (MPO) levels, cytokine concentrations, tight junction protein expression, and SCFA production were measured to evaluate treatment effects. Histological analyses assessed inflammation, crypt damage, and ulceration. Mice treated with *L. reuteri* exhibited significant improvements across all evaluated parameters. Supplementation mitigated weight loss, reduced DAI, and restored colon length. MPO levels and pro-inflammatory cytokines (TNF-α, IL-1β, IL-6) were significantly reduced, while anti-inflammatory IL-10 levels were elevated. Histological scores showed decreased inflammation, crypt damage, and ulceration. *L. reuteri* enhanced tight junction protein expression, particularly ZO-1 and Claudin-1, and significantly increased SCFA production, improving gut barrier integrity and microbial function. *L. reuteri* supplementation effectively mitigates DSS-induced colitis by reducing inflammation, restoring intestinal barrier integrity, and enhancing microbial metabolism. These findings suggest *L. reuteri* as a promising therapeutic candidate for UC management.

## 1. Introduction

Ulcerative colitis (UC) is a chronic inflammatory condition affecting the colorectal region, characterized by symptoms such as ulceration, abdominal pain, diarrhea, and rectal bleeding [1]. Although the precise etiology of UC remains unclear, mounting evidence suggests that an interplay of factors (including immune dysregulation, mucosal barrier damage, genetic predisposition, and environmental triggers) contributes to its pathogenesis [1,2]. The DSS-induced colitis model is a widely used experimental tool for studying UC due to its ability to replicate the clinical and histological features of the disease. DSS disrupts the gut epithelial barrier, leading to acute inflammation marked by granulocyte infiltration, ulceration, and mucosal damage [3].

Recent advances in bioinformatics and gut microbiota research have highlighted the critical role of intestinal microorganisms in the onset and progression of UC [4]. The gut microbiota plays an essential role in maintaining the intestinal mucosal barrier, a vital defense mechanism against pathogens [5,6]. Comprising mechanical, chemical, biological, and immune components, the intestinal barrier is maintained by a single layer of columnar epithelial cells connected by tight junctions [7]. Disruptions in gut microbial composition, commonly observed in UC, can compromise this barrier and exacerbate inflammation [8]. Advances in sequencing technologies have provided deeper insights into the gut ecosystem, further supporting its role in UC pathogenesis [6].

Importantly, lactic acid bacteria (LAB), including species such as *Lactobacillus reuteri*, contribute significantly to the defense against pathogenic microorganisms by producing antimicrobial substances like lactic acid, reuterin, and bacteriocins [9]. These compounds inhibit the growth of various intestinal pathogens, including *Escherichia coli*, *Clostridium difficile*, *Salmonella enterica*, and *Helicobacter pylori* [10]. By competing with these pathogenic bacteria for adhesion sites and nutrients, LAB help maintain a balanced gut microbiota and protect intestinal health [11].

Conventional treatments for UC, including aminosalicylates, corticosteroids, immunomodulators, and biologics, have shown varying degrees of effectiveness but are often accompanied by adverse side effects such as infections, allergies, and organ toxicity [12]. Aminosalicylates like mesalamine are commonly used to reduce inflammation but can cause side effects such as headache, abdominal pain, and, in rare cases, kidney toxicity [13]. Corticosteroids, while effective for inducing remission, carry risks including weight gain, osteoporosis, hyperglycemia, and increased susceptibility to infections [14]. Immunomodulators such as azathioprine and 6-mercaptopurine may lead to bone marrow suppression, liver toxicity, and a heightened risk of infections [15]. Biologic agents, including infliximab and adalimumab, have transformed UC management but are associated with adverse effects like infusion reactions, opportunistic infections, and the development of anti-drug antibodies, which can reduce therapeutic efficacy over time [16]. These limitations underscore the need for alternative, safer therapeutic options.

Probiotics, particularly *L. reuteri*, have emerged as promising candidates due to their strong safety profile, minimal side effects, and beneficial roles in modulating gut microbiota and immune responses [17]. While probiotics are generally considered safe for healthy individuals, rare cases of infections have been reported in severely immunocompromised patients; however, their overall safety compared to conventional drugs makes them attractive therapeutic options [17]. Moreover, *L. reuteri* is known not only for its immunomodulatory and anti-inflammatory properties but also for its ability to enhance short-chain fatty acid (SCFA) production, including acetic acid and butyric acid, which play crucial roles in maintaining intestinal barrier integrity, regulating immune function, and promoting epithelial health [18]. Numerous animal studies and clinical trials have demonstrated the protective effects of *L. reuteri* against colitis and its potential role in reducing the risk of inflammatory bowel disease (IBD) [19–21].

This study aims to explore the preventive and mechanistic actions of *L. reuteri*, using a strain isolated from human breast milk, in a DSS-induced murine model of colitis by assessing comprehensive parameters, including histopathological alterations, inflammatory cytokine levels, and intestinal barrier function, which have not been simultaneously addressed in previous studies.

## 2. Materials and methods

### 2.1. Culturing and quantification of *Lactobacillus* *reuteri*

*L. reuteri,* initially derived from human breast milk, was supplied by Parsi Lact® (Pardis Roshd Mehregan, Shiraz, Iran) under strain code PLR-01. For taxonomic confirmation, the strain was identified as *L. reuteri* via 16S rRNA gene sequencing performed by the supplier. In brief, genomic DNA was extracted and the 16S rRNA gene was amplified using universal primers 27F (5^′^-AGAGTTTGATCCTGGCTCAG-3^′^) and 1492R (5^′^-GGTTACCTTGTTACGACTT-3^′^) [22]. The resulting sequence was analyzed using BLAST against the NCBI database, confirming >99% sequence similarity with *L. reuteri* reference strains. Sanger sequencing was performed by Pishgam (Tehran, Iran), and the resulting 16S rDNA amplicon sequence was aligned against the GenBank nucleotide database using the BLAST algorithm, confirming over 99% sequence similarity with *L. reuteri* reference strains.

The bacteria were cultured anaerobically in De Man, Rogosa, and Sharpe (MRS) medium (Merck, Germany) following established protocols [23]. The initial culture was incubated at 37°C for 24 hours. After the initial incubation, the cultures were transferred to MRS agar plates using specific serial dilutions. The plates were incubated under anaerobic conditions at 37°C for 48–72 hours to promote colony development. To establish a standard curve for colony-forming units (CFU) per milliliter, the optical density at 600 nm (OD600) was measured for bacterial suspensions cultured on MRS agar at predetermined concentrations.

Quantitative analysis of bacterial growth in the culture medium was performed by comparing the OD600 values of the samples to the established standard curve, enabling precise determination of bacterial concentrations.

### 2.2. Animals

This study was approved by the Ethics Committee of Hamadan University of Medical Sciences (Ethical approval code: IR.UMSHA.REC.1401.866) and conducted in accordance with institutional and national regulations for the care and use of laboratory animals. The study adhered to the ARRIVE guidelines to ensure ethical and methodological rigor, and all procedures were carried out by trained personnel with expertise in animal handling, anesthesia, and euthanasia [24].

A total of 24 male Balb/C mice, aged 6–8 weeks and weighing between 25 and 30 g, were obtained from Hamadan University of Medical Sciences. The mice were individually housed in sterile cages under a controlled 12-hour light/dark cycle with unrestricted access to standard chow and water. The mice were individually housed in sterile cages under a controlled 12-hour light/dark cycle with unrestricted access to standard chow and water. To ensure physiological stability, they underwent a two-week acclimatization period before the initiation of experimental procedures.

To establish an experimental colitis model, 3% dextran sulfate sodium (DSS) (Sigma-Aldrich, USA) with a molecular weight of 5000 daltons was dissolved in drinking water and administered daily for one week. Following colitis induction, the mice were randomly assigned to three experimental groups (n = 8 per group) to evaluate the effects of *L. reuteri* on colitis progression and treatment outcomes.

The normal control group received neither DSS nor probiotic treatment and served as a baseline for assessing physiological parameters. The DSS control group was exposed to DSS without any probiotic or drug intervention, serving as a model for colitis induction and disease progression. The DSS + *L. reuteri* 10^10^ group received daily oral gavage of *L. reuteri* at a concentration of 10^10^ CFU in 100 μl of de MRS medium, administered concurrently with DSS exposure. All treatment groups received daily oral gavage for 21 days (from day −14 to day 7), with probiotics being administered as specified. Mice in the normal control group and DSS groups received saline gavage for the same duration (Table 1).

**Table 1:** Gavage schedule and treatment protocol for all experimental groups.

Group	Treatment	Gavage Schedule	Duration of Treatment
**Normal Control**	No DSS or probiotic treatment	Saline gavage	Day −14 to day 7
**DSS Control**	DSS exposure only	Saline gavage	Day −14 to day 7
**DSS + *L. reuteri* 10¹**⁰****	DSS + *L. reuteri* 10¹**⁰** CFU oral gavage	*L. reuteri* 10¹**⁰** CFU in 100 μl of MRS medium, daily	Day −14 to day 7

To ensure animal welfare, mice were monitored twice daily throughout the experiment for body weight changes, with any loss exceeding 20% of initial weight considered a humane endpoint. Clinical signs of colitis, including severe diarrhea, dehydration (skin tenting, sunken eyes), lethargy, and labored breathing, were closely observed along with general behavior and distress levels. None of the animals reached the humane endpoint criteria during the study. At the conclusion of the study, all mice were euthanized using CO₂ inhalation followed by cervical dislocation to confirm complete cessation of vital functions. Prior to euthanasia, mice were anesthetized via intraperitoneal injection of ketamine (80 mg/kg) and xylazine (10 mg/kg) to ensure a painless procedure. All experimental procedures, including animal handling, DSS administration, probiotic treatment, monitoring, anesthesia, and euthanasia, were conducted by trained personnel in accordance with ethical guidelines and institutional regulations [25].

### 2.3. Disease activity index (DAI) scoring

The disease activity index (DAI) was assessed using a scoring system that incorporated three parameters: body weight change, stool consistency, and hemoccult bleeding, based on a previously established method [26]. Each parameter was scored individually, and the final DAI score was calculated as the average of these three scores.

Body weight change was evaluated on a scale from 0 to 6, with scores assigned as follows: 0 for no weight loss (<0%), 1 for a weight loss of 0–5%, 2 for 6–10%, 3 for 11–15%, 4 for 16–20%, 5 for 21–25%, and 6 for 26–30%. This parameter accounted for the degree of weight loss as a quantitative indicator of disease severity.

Stool consistency was assessed using a scoring system ranging from 0 to 3. Mice with normal stools received a score of 0, while soft stools were rated as 1, loose stools as 2, and watery stools as 3. This scoring method enabled the assessment of diarrhea severity, a key indicator of colitis.

Hematochezia (the presence of blood in stool) was assessed using a grading scale from 0 to 3. A score of 0 represented no detectable occult blood in the feces, while a score of 1 indicated minor blood streaks. A score of 2 was assigned when feces were visibly coated with blood, and a score of 3 denoted noticeable rectal bleeding. This evaluation served as an indicator of intestinal bleeding linked to colitis.

By averaging the scores for body weight change, stool consistency, and hemoccult bleeding, the DAI provided a comprehensive indicator of disease activity in experimental models of colitis.

### 2.4. Sample collection and colon length measurement

Following euthanasia, the entire colon was carefully removed and its length was immediately measured to assess potential changes associated with colitis severity. After measurement, the colon tissue and its contents were collected and stored at −80°C for subsequent analyses.

Additionally, blood samples were collected via cardiac puncture under sterile conditions. The collected blood was allowed to coagulate at room temperature before serum separation by centrifugation at 3,000 g for 20 minutes. The serum was then stored at −80°C to preserve its integrity for future biochemical analyses.

### 2.5. Myeloperoxidase (MPO) analysis

Myeloperoxidase (MPO) levels, a key marker of neutrophil infiltration and inflammation, were assessed to evaluate the inflammatory response in the colon tissue [27]. Test kits specifically designed for MPO analysis were procured from Kiazist Co. Ltd (Cat. No. KiaZist-MPO-96T). The analysis was performed according to the manufacturer’s guidelines to ensure accurate and reliable results. This assessment provided critical insights into the extent of inflammation in the colon tissue of the experimental model.

### 2.6. Histological analysis

The histological analysis followed the method described in previous research [28]. Briefly, distal colon samples were preserved in a 4% paraformaldehyde solution for 24 hours. After fixation, the samples were embedded in paraffin and sectioned into thin slices measuring 5 μm in thickness. The sections were then stained with hematoxylin and eosin (H&E) for microscopic evaluation.

The stained sections were examined under a microscopy (BX41, Olympus) at magnifications of ×40 and ×200 to assess histological changes. The severity of colonic injury was evaluated using a scoring system that considered three parameters: the degree of inflammation (scored from 0 to a maximum of 3), crypt damage (scored from 0 to a maximum of 5), and the presence of ulcerations (scored from 0 to a maximum of 3). This scoring system followed the method outlined by Laroui et al. (2012), enabling a comprehensive assessment of histological damage in the colonic tissue.

### 2.7. Measurement of cytokine levels in colon tissues

Cytokine levels in colon tissues were determined following methods described in previous studies. Colon tissues were homogenized in PBS buffer containing protease inhibitor cocktail (Roche, Germany) using a tissue homogenizer (IKA T25 digital ULTRA-TURRAX, Germany). The homogenates were then centrifuged at 4°C to separate the supernatant, which was collected for cytokine analysis.

The concentrations of key cytokines, including tumor necrosis factor-alpha (TNF-α), IL-1β, IL-6, and IL-10, were measured using enzyme-linked immunosorbent assay (ELISA) test kits purchased from Carmania Parsgen Co. The assays were performed according to the manufacturer’s protocols.

### 2.8. Quantitative real-time PCR analysis

Quantitative real-time PCR (qRT-PCR) was utilized to examine the expression of specific genes in colon tissue samples [29]. Total RNA was isolated from the tissues using the Total RNX-Plus kit (SinaColon Co, Iran), following the manufacturer’s instructions. A spectrophotometer was employed to evaluate RNA quality and concentration, ensuring sample integrity. The extracted RNA was then used to synthesize complementary DNA (cDNA) with the cDNA Synthesis kit (Parstous Biotechnology, Iran). The reverse transcription process was performed under optimized conditions to convert RNA into stable cDNA, suitable for amplification and analysis. Gene expression analysis was conducted using the GoTaq R SYBR Green qPCR Master Mix (SinaColon Co, Iran) in a qRT-PCR system. Primers specific to the target genes, including ZO-1, Claudin-1, and Occludin, were synthesized by Pishgam Co., Ltd. (Iran). The primer sequences and details are listed in Table 2. GAPDH was used as the housekeeping gene to normalize the data and account for variations in RNA quantity and cDNA synthesis efficiency. The qRT-PCR (Qiagen, Germany) reactions were carried out in a 96-well plate format, and fluorescence data were collected at the end of each cycle to monitor the amplification process. Relative gene expression levels were calculated using the 2^−ΔΔCt^ method, comparing the expression levels of target genes to those of the housekeeping gene [30].

**Table 2:** Detailed primer sequences utilized for qRT-PCR analysis.

Gene	Forward Primer (F) (5′-3′)	Reverse Primer (R) (5′-3′)
**Claudin-1**	AAGACGACGAGGCACAGAAGA	GAAGGTGCTGGCTTGGGATAG
**Occludin**	CAGCAGCAGTGGTAACTTGGA	CCGGTCGTGTAGTCTGTTTCAT
**ZO-1**	GCGAAATGAGAAACAAGCACC	ATGAGTTGAGTTGGCAGGAC
**GAPDH**	GTTTGTGATGGGCGTGAACC	CAGTCTTCTGGGTGGCAGTGAT

### 2.9. Analysis of short-chain fatty acid (SCFA) content

The quantification of short-chain fatty acids (SCFAs) was conducted following the method outlined in previous research [31]. Approximately 80 mg of cecal contents were processed using a HALO-F100 fecal analyzer (Aosheng, China). For the analysis, 500 µl of the sample was mixed with crotonate monophosphate solution to enhance detection. The mixture was subsequently filtered through a 0.22 µm syringe filter (Sartorius, Germany), and the resulting supernatant was transferred into a gas-tight vial.

SCFA concentrations were measured using gas chromatography (GC) on an Agilent 7890B GC system (Agilent Technologies, USA) equipped with a flame ionization detector (FID). Separation was achieved using a HP-FFAP capillary column (30 m × 0.32 mm i.d. × 0.25 µm film thickness; Agilent Technologies). The injector and detector temperatures were set at 250°C and 280°C, respectively. Helium was used as the carrier gas at a flow rate of 1.5 mL/min. The oven temperature program was as follows: initial temperature at 100°C held for 2 min, increased at 8°C/min to 180°C, and then at 20°C/min to 240°C, held for 5 min. The injection volume was 1 µl in splitless mode.

### 2.10. Statistical analysis

Data were analyzed using GraphPad Prism (version 10.4.1). For comparisons among multiple groups, one-way ANOVA followed by Tukey’s post hoc test was employed. Results are presented as mean ± SD, and p < 0.05 was considered statistically significant. This statistical approach was applied consistently to assess differences in colitis-associated parameters, cytokine levels, tight junction protein expression, and SCFA concentrations, providing a robust basis for evaluating the protective effects of *L. reuteri* in DSS-induced colitis.

## 3. Results

### 3.1. Effects of *Lactobacillus reuteri* on DSS-induced colitis symptoms

**Fig 1:**
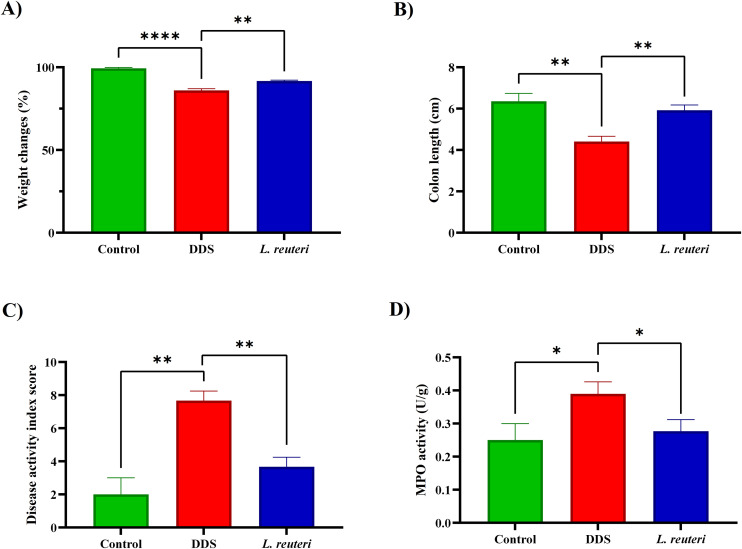
This figure illustrates the impact of *Lactobacillus reuteri* supplementation on key indicators of colitis induced by dextran sulfate sodium (DSS). **(A)** Changes in body weight (%) highlight the protective effect of *L. reuteri* against DSS-induced weight loss. **(B)** Colon length measurements demonstrate the restoration of colon length in mice treated with *L. reuteri* compared to the DSS-only group. **(C)** Disease activity index (DAI) scores indicate a significant reduction in colitis severity in the *L. reuteri*-supplemented group. **(D)** Myeloperoxidase (MPO) activity in colon tissue, expressed as units per gram (U/g), shows decreased inflammation levels with *L. reuteri* supplementation. The data are expressed as mean ± standard deviation (SD) for three biological replicates (n = 8). Statistical significance is denoted as follows: *p < 0.05, **p < 0.01, ***p < 0.001, and ****p < 0.0001.

Our findings revealed significant differences in colitis-associated parameters among experimental groups (Fig 1). DSS-exposed mice exhibited marked weight loss, colon shortening, and elevated disease activity compared to controls. Specifically, the DSS group showed a significant decrease in body weight (80.44% ± 5.48) compared to the control group (94.53% ± 12.59, p < 0.0001). *L. reuteri* supplementation mitigated weight loss, with treated mice retaining significantly more body weight (88.82% ± 4.31) than DSS mice (p = 0.0012), highlighting its protective effect.

DSS treatment induced significant colon shortening (4.46 ± 0.47 cm vs. control: 6.22 ± 0.56 cm, p < 0.0001). *L. reuteri* treatment partially restored colon length (5.45 ± 0.48 cm, p = 0.0022 vs. DSS), demonstrating alleviation of inflammation-induced tissue damage. Disease activity index (DAI) scores were also significantly elevated in DSS mice (7.71 ± 1.13) compared to controls (2.01 ± 1.11, p < 0.0001), while *L. reuteri* supplementation reduced DAI to 3.71 ± 0.95 (p < 0.0001), indicating reduced symptom severity.

Myeloperoxidase (MPO) activity, a marker of neutrophil infiltration, was elevated in DSS mice (0.405 ± 0.124 U/g) relative to controls (0.251 ± 0.161 U/g, p = 0.0045). *L. reuteri* significantly reduced MPO levels to 0.296 ± 0.124 U/g (p = 0.0082), suggesting attenuation of mucosal inflammation.

### 3.2. Effects of *Lactobacillus reuteri* on colon histology in DSS-induced colitis

**Fig 2:**
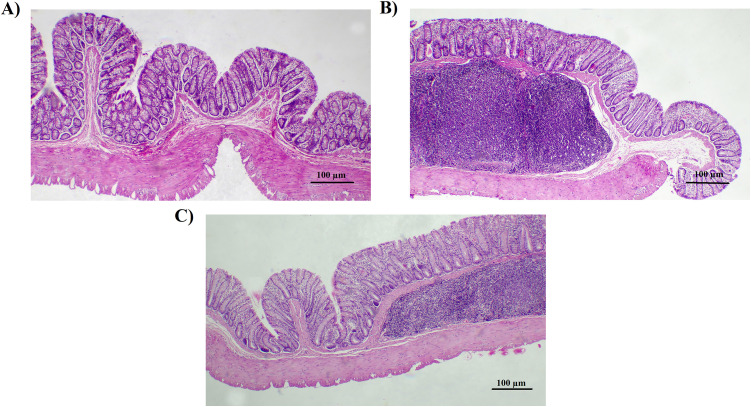
The figure illustrates the impact of *Lactobacillus reuteri* supplementation on colon histology, as observed through hematoxylin and eosin (H&E) staining of tissue sections at 100× magnification. **(A)** Control group with normal histological structure. **(B)** DSS-treated group with severe damage and inflammation. **(C)** *L. reuteri*-treated group with improved tissue integrity and reduced inflammation.

**Fig 3:**
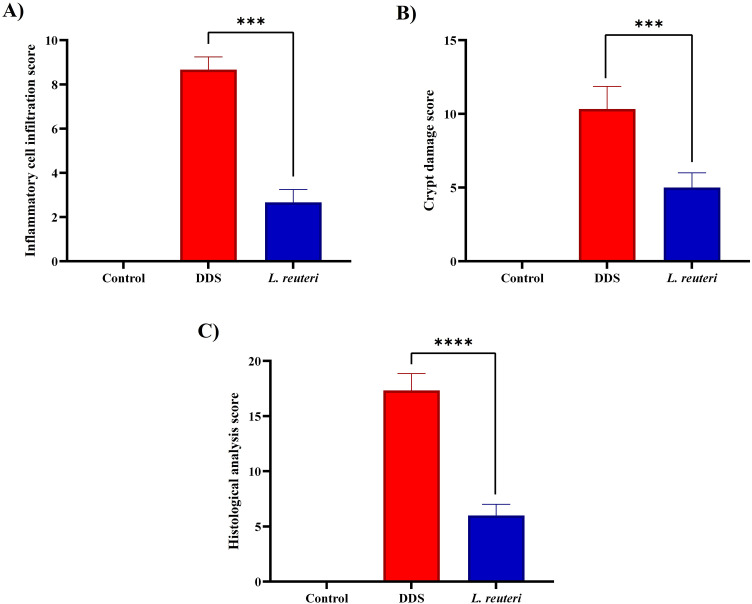
This figure illustrates key histological features associated with colitis severity and tissue damage. **(A)** The extent of inflammatory cell infiltration, highlighting the presence and severity of immune cell infiltration into the colonic tissue. **(B)** Crypt damage, depicting structural alterations and destruction of colonic crypts as a result of inflammation. **(C)** The overall histological analysis score, summarizing the cumulative impact of inflammatory infiltration and crypt damage. The data are presented as mean ± standard deviation (SD) based on three biological replicates (n = 8). Statistical significance is indicated as follows: ***p < 0.001 and ****p < 0.0001.

H&E staining revealed that DSS treatment caused crypt destruction, mucosal erosion, and inflammatory cell infiltration, while *L. reuteri* markedly improved tissue integrity, reduced immune cell infiltration, and partially restored crypt architecture (Figs 2 and 3). Inflammatory cell infiltration scores decreased from 8.67 ± 0.58 (DSS) to 2.67 ± 0.61 with *L. reuteri* (p = 0.0002). Crypt damage scores declined from 10.22 ± 0.97 to 5.00 ± 0.82 (p = 0.0003). The combined histological score reduced from 17.14 ± 1.07 (DSS) to 6.29 ± 1.11 (p < 0.0001), confirming substantial protection of mucosal integrity.

Inflammatory cell infiltration scores (Fig 3A) were significantly elevated in the DSS group (8.67 ± 0.58) compared to the control group (0 ± 0). However, *L. reuteri* treatment markedly reduced the infiltration score to 2.67 ± 0.61 (*p* = 0.0002), indicating a strong anti-inflammatory effect.

Crypt damage scores (Fig 3B) followed a similar trend. While the control group had no detectable damage (0 ± 0), the DSS group showed a mean crypt damage score of 10.22 ± 0.97. This score significantly decreased to 5.00 ± 0.82 in the *L. reuteri*-treated group (*p* = 0.0003), demonstrating the protective role of *L. reuteri* in maintaining crypt structure.

The total histological analysis score (Fig 3C), which combines measures of inflammatory cell infiltration and structural tissue damage, was 17.14 ± 1.07 in the DSS group and 0 ± 0 in the control group. Notably, *L. reuteri* treatment significantly reduced this overall score to 6.29 ± 1.11 (*p* < 0.0001), indicating substantial preservation of mucosal integrity.

### 3.3. Impact of *Lactobacillus reuteri* on cytokine levels in colon tissue

**Fig 4:**
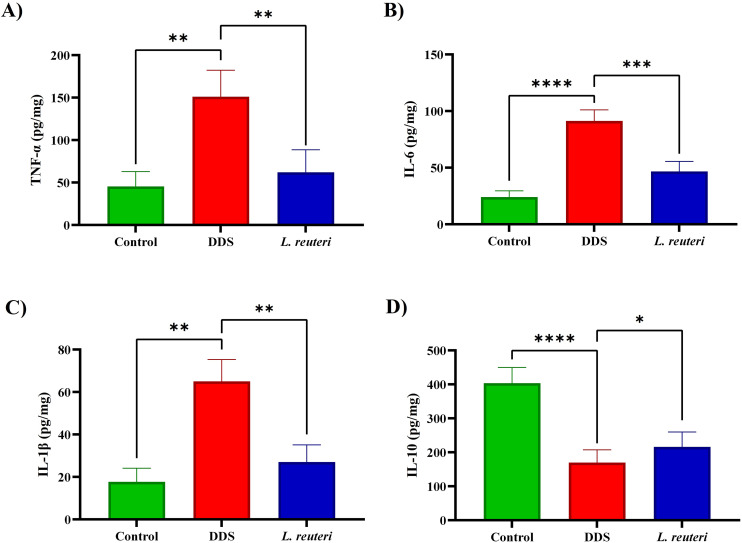
This figure illustrates the impact of *Lactobacillus reuteri* supplementation on cytokine concentrations in a mouse model of colitis induced by dextran sulfate sodium (DSS). **(A)** Tumor necrosis factor-alpha (TNF-α): Pro-inflammatory cytokine levels were significantly elevated in the DSS group but were notably reduced in the group treated with *L. reuteri*. **(B)** Interleukin-1 beta (IL-1β): Similarly, IL-1β levels, heightened in the DSS group, showed a marked decrease following *L. reuteri* supplementation. **(C)** Interleukin-6 (IL-6): DSS treatment caused a significant increase in IL-6 levels, which were significantly mitigated in the *L. reuteri*-treated group. **(D)** Interleukin-10 (IL-10): The anti-inflammatory cytokine IL-10, reduced in the DSS group, was significantly elevated with *L. reuteri* supplementation. Data are presented as mean ± standard deviation (SD) based on three biological replicates (n = 8). Statistical significance is denoted as follows: *p < 0.05, **p < 0.01, ***p < 0.001, and ****p < 0.0001.

The administration of *L. reuteri* significantly modulated cytokine production in the colonic tissue of mice with DSS-induced colitis. As shown in Fig 4, pro-inflammatory cytokines including IL-1β, IL-6, and TNF-α were markedly elevated in the DSS group compared to the control, indicating an intensified inflammatory response. Treatment with *L. reuteri* effectively attenuated these elevations, highlighting its immunomodulatory potential. Specifically, TNF-α levels increased to 149.32 ± 37.24 pg/mg in the DSS group versus 45.34 ± 17.04 pg/mg in controls, whereas *L. reuteri* reduced TNF-α to 62.01 ± 18.72 pg/mg (p = 0.0091), corresponding to a 58.9% decrease. Similarly, IL-6 rose to 91.43 ± 6.73 pg/mg in DSS mice compared to 24.12 ± 5.57 pg/mg in controls, and supplementation with *L. reuteri* significantly lowered it to 47.62 ± 5.53 pg/mg (p = 0.0003), reflecting a 48.9% reduction. IL-1β levels also increased from 17.67 ± 15.43 pg/mg in controls to 64.87 ± 17.81 pg/mg in the DSS group and were effectively reduced to 26.91 ± 16.36 pg/mg following *L. reuteri* administration (p = 0.0022, 58.5% decrease). Conversely, the anti-inflammatory cytokine IL-10 was suppressed in DSS-treated mice (169.67 ± 36.12 pg/mg) compared to controls (403.67 ± 38.93 pg/mg, p < 0.0001), and *L. reuteri* significantly restored IL-10 levels to 215.97 ± 34.11 pg/mg (p = 0.0271).

### 3.4. Effects of *Lactobacillus reuteri* on intestinal barrier function

**Fig 5:**
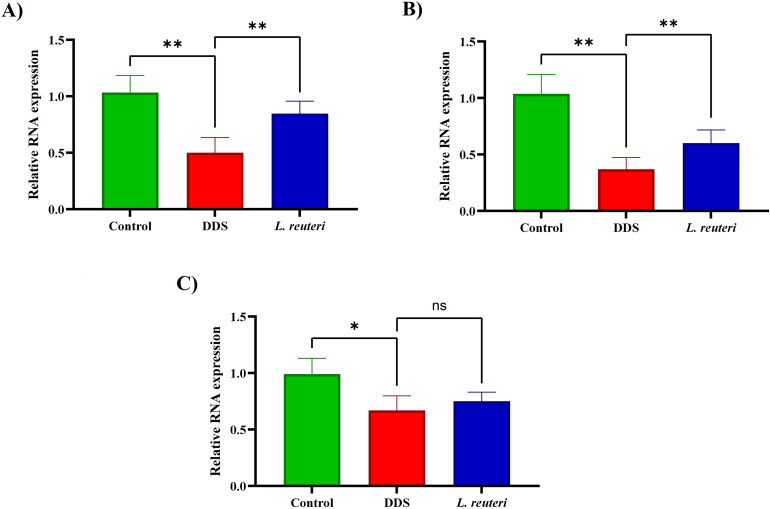
Effect of *Lactobacillus reuteri* supplementation on the expression of intestinal barrier-related genes in colonic tissues. **(A)** ZO-1, **(B)** Claudin-1, **(C)** Occludin. Data are presented as mean ± standard deviation (SD) based on three biological replicates (n = 8). Significance is indicated as follows: *p < 0.05, and **p < 0.01.

DSS caused significant reduction in tight junction proteins: ZO-1 (p = 0.0014), Claudin-1 (p = 0.0030), and Occludin (p = 0.0202). *L. reuteri* supplementation significantly increased ZO-1 (p = 0.001) and Claudin-1 (p = 0.0034), with a non-significant upward trend in Occludin (p = 0.0647), suggesting preservation of intestinal barrier integrity (Fig 5).

### 3.5. Effects of *Lactobacillus reuteri* on SCFAs production

**Fig 6:**
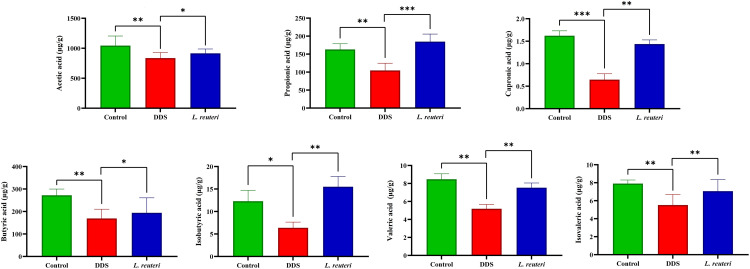
Effects of Lactobacillus reuteri on short-chain fatty acid (SCFA) levels across experimental groups. The figure depicts the concentrations of various SCFAs, including acetic acid, propionic acid, caproic acid, butyric acid, isobutyric acid, valeric acid, and isovaleric acid, across three groups: the control group, the DSS-induced colitis group, and the L. reuteri supplementation group. The data are presented as mean ± standard deviation (SD) based on three biological replicates (n = 8). Statistical significance is indicated as follows: *p < 0.05, **p < 0.01, and ***p < 0.001.

SCFAs play a crucial role in maintaining host health by serving as an energy source, supporting intestinal epithelial barrier integrity, and mitigating inflammation. In our study, DSS treatment significantly altered SCFA levels, whereas supplementation with *L. reuteri* effectively restored these metabolites (Fig 6).

Acetic acid levels were significantly reduced in the DSS group (837.33 ± 125.16 μg/g) compared to the control group (1044.67 ± 247.62 μg/g, *p* = 0.0037). Administration of *L. reuteri* partially restored these levels to 915.67 ± 86.52 μg/g (*p* = 0.0230), indicating an ameliorative effect.

Propionic acid also exhibited a marked decline following DSS treatment, with concentrations falling to 104.67 ± 26.03 μg/g, significantly lower than those in control mice (162.89 ± 22.10 μg/g, *p* = 0.0040). Supplementation with *L. reuteri* significantly elevated propionic acid levels to 184.67 ± 25.12 μg/g (*p* = 0.0003), surpassing even the levels observed in the control group.

Caproic acid levels were also suppressed by DSS (0.65 ± 0.13 μg/g) relative to the control (1.62 ± 0.11 μg/g, *p* = 0.0006). Supplementation with *L. reuteri* significantly restored its concentration to 1.44 ± 0.09 μg/g (*p* = 0.0010).

Butyric acid, a critical SCFA with well-established roles in epithelial health and immune regulation, was significantly diminished in the DSS group (169.07 ± 31.07 μg/g) compared to the control (272.12 ± 29.31 μg/g, *p* = 0.0014). *L. reuteri* administration increased its concentration to 193.97 ± 42.39 μg/g (*p* = 0.0310), indicating a partial recovery.

Isobutyric acid was substantially decreased in DSS-treated mice (6.37 ± 1.72 μg/g) relative to controls (12.32 ± 2.85 μg/g, *p* = 0.0145). Treatment with *L. reuteri* restored the concentration to 15.13 ± 2.52 μg/g (*p* = 0.0026), suggesting normalization of microbial fermentative activity.

Valeric acid levels in the DSS group (5.19 ± 1.47 μg/g) were significantly lower than in controls (8.47 ± 2.07 μg/g, *p* = 0.0019). *L. reuteri* treatment increased these levels to 7.53 ± 1.74 μg/g (*p* = 0.0047), reflecting a notable recovery.

Finally, Isovaleric acid levels decreased in DSS-treated mice (5.52 ± 2.84 μg/g) in comparison to the control group (7.92 ± 0.88 μg/g, *p* = 0.0027). Treatment with *L. reuteri* significantly increased its levels to 7.06 ± 2.32 μg/g (*p* = 0.0058), approaching normal values.

## 4. Discussion

UC is a chronic IBD that has been rapidly increasing in prevalence, particularly among younger populations. These include changes in environmental exposures, such as increased urbanization, westernized dietary patterns high in fat and low in fiber, altered gut microbiota composition, and improved hygiene practices that may influence immune system development [32]. This rising incidence poses significant challenges in managing the disease, as UC is characterized by high recurrence rates and limited long-term treatment options with varying degrees of efficacy [33]. These challenges have driven growing interest in alternative therapies, such as probiotics, that could potentially modulate intestinal inflammation and restore gut homeostasis. Among these, *L. reuteri* has garnered attention for its promising effects in regulating the gut environment and modulating immune responses [34]. The current study sought to investigate the protective effects of *L. reuteri* in a DSS-induced colitis mouse model, focusing on its influence on intestinal barrier function, inflammatory responses, and SCFA production. The findings from this study provide compelling evidence for the beneficial effects of *L. reuteri* in managing UC pathophysiology.

The DSS-induced colitis model effectively replicates several key pathological features of UC, including significant weight loss, diarrhea, hematochezia, and the elevation of markers such as DAI and MPO, which are indicative of inflammation [35]. In our experiments, DSS exposure resulted in notable weight loss, increased DAI and MPO levels, shortened colon length, and substantial tissue damage. These findings confirmed that DSS successfully induced colitis, making it a reliable model for studying UC. These results are similar to findings by Ahmad et al., who reported comparable reductions in colon length and increases in MPO activity in DSS-treated mice, supporting the validity of our model [36]. Supplementation with *L. reuteri* significantly alleviated these effects. *L. reuteri* not only restored colon length but also reduced inflammation and promoted tissue repair, demonstrating its protective properties against DSS-induced damage. These results are consistent with previous studies by Dicksved et al. and Lin et al., which reported that *L. reuteri* improves UC symptoms by enhancing intestinal barrier integrity, modulating gut microbiota, and suppressing inflammatory cytokine pathways [37,38].

One of the key mechanisms underlying colon inflammation in UC is the dysregulation of cytokine production, particularly the imbalance between pro-inflammatory cytokines, such as TNF-α, IL-1β, and IL-6, and the protective anti-inflammatory cytokine IL-10 [39,40]. In our study, we observed that DSS exposure induced a significant increase in pro-inflammatory cytokines, which was accompanied by a marked reduction in IL-10 levels. This shift in the cytokine profile contributes to the worsening of mucosal damage and exacerbates the inflammatory response, further advancing the disease process. However, supplementation with *L. reuteri* appeared to reverse this imbalance, as it effectively suppressed the levels of pro-inflammatory cytokines while simultaneously boosting the expression of IL-10. Taken together with prior findings from Whary et al., these results suggest that *L. reuteri* consistently exerts an immunomodulatory effect across different models of IBD, thereby reinforcing the biological plausibility of its anti-inflammatory action in UC [41].

Furthermore, although the current study did not directly evaluate molecular signalling pathways, existing literature suggests that *L. reuteri* exerts its protective effects through the modulation of key inflammatory pathways, including the nuclear factor kappa B (NF-κB) and mitogen-activated protein kinase (MAPK) pathways [42]. The NF-κB signalling pathway is a central mediator of inflammatory responses, regulating the transcription of various pro-inflammatory cytokines [43]. Probiotic strains like *L. reuteri* have been reported to inhibit NF-κB activation, thereby reducing inflammation. Similarly, the MAPK pathway, which plays a role in cellular responses to stress and cytokines, is also modulated by probiotics, contributing to the suppression of intestinal inflammation [44]. Additionally, *L. reuteri* may influence toll-like receptor (TLR) signalling, which is crucial for host-microbial interactions and immune homeostasis [45]. By integrating our cytokine and SCFA results with these known signalling pathways, our study helps explain how *L. reuteri* simultaneously reduces inflammation and supports mucosal healing, providing a mechanistic bridge between clinical outcomes and molecular regulation.

The intestinal epithelial barrier plays a crucial role in regulating gut permeability and maintaining immune homeostasis. This barrier is maintained by tight junction proteins such as ZO-1, Occludin, and Claudins, which, when damaged, lead to increased intestinal permeability and exacerbate inflammation, hallmarks of colitis [46,47]. In our study, we found that *L. reuteri* supplementation significantly upregulated the expression of ZO-1 and Claudin-1, while Occludin showed a non-significant increase. These changes collectively suggest improved intestinal barrier integrity and a reduction in inflammation, supporting the idea that *L. reuteri* plays a critical role in preserving gut barrier function and mitigating colitis-induced damage. Compared to other probiotics such as *Bifidobacterium longum* and *Lactobacillus rhamnosus*, which only modestly improved barrier integrity, our findings highlight *L. reuteri* as a potentially more potent candidate for restoring tight junction protein expression and maintaining epithelial resilience [48,49].

SCFAs, including acetic acid, propionic acid, and butyric acid, are essential metabolites produced by gut microbiota that support intestinal health by regulating immune responses and maintaining epithelial barrier integrity [50,51]. Our study confirmed that DSS exposure led to a significant depletion of SCFAs, contributing to increased inflammation and impaired gut function. However, supplementation with *L. reuteri* restored SCFA levels, countering the adverse effects of DSS and promoting microbial activity in the gut. This finding is particularly significant because, unlike previous studies that reported only partial SCFA restoration, we observed a near-complete recovery of butyrate levels, a metabolite strongly linked with epithelial energy supply and anti-inflammatory action. These data integrate well with the broader literature and point toward SCFA recovery as a central mechanism through which *L. reuteri* confers its protective effects in UC [52].

## 5. Conclusion

This study demonstrates that *L. reuteri* holds significant therapeutic potential in alleviating the pathological effects of DSS-induced colitis. Our findings show that *L. reuteri* can reduce inflammation, strengthen intestinal barrier integrity, and restore SCFA production, suggesting that it could be an effective adjunct or alternative to current treatments for ulcerative colitis. While several clinical trials are underway to evaluate the benefits of *L. reuteri* in patients with UC and other inflammatory bowel diseases, large-scale randomized controlled trials remain limited. The results from existing studies are promising, indicating improvements in clinical symptoms and reductions in inflammatory markers following *L. reuteri* supplementation. However, further clinical validation is necessary to establish standardized treatment protocols. Future research should include gut microbiota profiling, extended post-treatment follow-up, and direct investigation of signaling pathways to better understand the mechanisms underlying the effects of *L. reuteri*. Additionally, determining the optimal dosage, treatment duration, and timing of administration will be essential to maximize its therapeutic benefits. Identifying specific *L. reuteri* strains with superior properties could also enhance the precision of probiotic interventions. Exploring its combination with other probiotics, prebiotics, or novel biologic therapies may yield synergistic effects, offering improved anti-inflammatory actions and mucosal healing. Finally, long-term studies assessing safety and sustained efficacy are needed to support the integration of *L. reuteri* into evidence-based clinical practice for managing inflammatory bowel disease.
